# MYD88 L265P Mutations Are Correlated with 6q Deletion in Korean Patients with Waldenström Macroglobulinemia

**DOI:** 10.1155/2014/363540

**Published:** 2014-05-07

**Authors:** Jung-Ah Kim, Kyongok Im, Si Nae Park, Jiseok Kwon, Qute Choi, Sang Mee Hwang, Naohiro Sekiguchi, Sung-Soo Yoon, Dong Soon Lee, Seon Young Kim

**Affiliations:** ^1^Department of Laboratory Medicine, Seoul National University College of Medicine, 101 Daehangno, Jongno-gu, Seoul 110-744, Republic of Korea; ^2^Cancer Research Institute, Seoul National University College of Medicine, Seoul, Republic of Korea; ^3^Department of Laboratory Medicine, Seoul National University Bundang Hospital, Seongnam, Republic of Korea; ^4^Hematology Division, National Hospital Organization Disaster Medical Center, Tokyo, Japan; ^5^Department of Internal Medicine, Seoul National University College of Medicine, Seoul, Republic of Korea

## Abstract

Waldenström macroglobulinemia (WM) is a malignant lymphoplasma-proliferative disorder with IgM monoclonal gammopathy. A recent whole-genome study identified MYD88 L265P as the key mutation in WM. We investigated *MYD88* mutations in conjunction with cytogenetic study in 22 consecutive Korean WM patients. Conventional G-banding and interphase fluorescence *in situ* hybridization (FISH) were performed at regions including 6q21 using bone marrow (BM) aspirates. Sixteen patients were subjected to Sanger sequencing-based *MYD88* mutation study. Five patients (28%) showed cytogenetic aberrations in G-banding. The incidence of 6q21 deletion was 17% by conventional G-banding and 37% by FISH. Ten patients (45%) showed cytogenetic aberrations using FISH: 6q deletion in eight (37%) and *IGH* rearrangement in four (18%). Two patients had both the 6q deletion and *IGH* rearrangement, and two had only the *IGH* rearrangement. Eleven patients (69%) presented with the MYD88 L265P mutation. *MYD88* mutations were significantly associated with the presence of 6q deletions (*P* = 0.037). Six patients with the 6q deletion for whom sequencing was possible were found to harbor *MYD88* mutations. The MYD88 L265P mutation was also associated with increased lymphocyte burden in BM biopsy. This is the first report of high frequency MYD88 L265P mutations in Korean WM patients.

## 1. Introduction

Waldenström macroglobulinemia (WM) is a malignant lymphoplasma-proliferative disorder characterized by bone marrow infiltration with lymphoplasmacytic lymphoma (LPL) and the secretion of monoclonal immunoglobulin M (IgM) [1–3]. Different stage B-lineage cells infiltrate into the bone marrow (BM), including small lymphocytes, lymphoplasmacytoid cells, and plasma cells [4]. Consensus recommendations from the Second International Workshop on WM diagnostic criteria require LPL with BM involvement and any concentration of IgM monoclonal gammopathy. The incidence rates for WM were 3.4/1,000,000 person-years among men and 1.7/1,000,000 person-years among women from 1988 to 1994 in the United States [5]. Meanwhile, the incidence of LPL in Korean individuals was reported to be low (0.8%) [6], although official data from the Korean national cancer registry are not available [7].

The genetic changes associated with WM are not fully elucidated. Familial clustering in WM suggests the occurrence of inherited genetic susceptibility, similar to chronic lymphocytic leukemia and multiple myeloma [2, 7]. Recently, a whole-exome sequencing study revealed a high frequency of the L265P mutation in the myeloid differentiation primary response gene 88 (*MYD88*) in WM [8]. In that study, 91% of LPL patients exhibited the MYD88 L265P mutation in their tumor cells. Depending on the sensitivity of detection method used, the reported incidence of MYD88 L265P ranges from 70% to 100% [8–14]. The* MYD88* gene, located on 3p22, encodes a cytosolic adapter protein that plays a central role in the innate and adaptive immune responses [15].

In the present study, we analyzed the MYD88 L265P mutation status of Korean WM patients using Sanger sequencing. We also investigated cytogenetic aberrations using conventional G-banding and fluorescence* in situ* hybridization (FISH) and analyzed their correlation with MYD88 L265P mutation status.

## 2. Materials and Methods

### 2.1. Patients

A series of 22 newly diagnosed WM patients treated at Seoul National University Hospital between December 2001 and November 2012 were included for this study. At first, 23 patients who were diagnosed as WM after BM study were selected retrospectively from the hospital information system. Among them, 22 patients whose BM samples were available for cytogenetic and molecular studies were finally included. WM was diagnosed according to the consensus recommendations from the Second International Workshop on WM [2] and the World Health Organization (WHO, 2008) classification criteria on LPL [3] using BM aspiration and biopsy specimens obtained at the time of diagnosis [3]. Each patient had IgM paraproteinemia and lymphoplasmacytic infiltration of the BM.

Mononuclear cells from the initial BM aspirates of all patients were fixed in Carnoy's solution and stored at −70°C for further cytogenetic analysis. The following laboratory and clinical information was obtained for each patient: date of diagnosis and start of therapy, age, sex, ethnicity, hemoglobin level, platelet count, and levels and type of paraprotein. Furthermore, we recorded the percentage of BM lymphocytes and plasma cell infiltration, performed conventional cytogenetic analyses of BM cells by G-banding, and assessed the presence of hepatosplenomegaly, lymphadenopathies, and the number of osteolytic lesions. All BM samples were collected with informed consent, and the study was reviewed and approved by the Institutional Review Board of Seoul National University College of Medicine.

### 2.2. Conventional Karyotyping by G-Banding

Conventional cytogenetic data were available for 18 of the 22 patients. Cytogenetic studies using standard G-banding techniques on heparinized BM samples were performed as part of the diagnostic workup. At least 20 metaphases were analyzed whenever possible. Clonal abnormalities were defined as two or more cells with the same chromosomal gain or structural rearrangement or at least three cells with the same chromosome deletion. Karyotypes were recorded according to the International System for Human Cytogenetic Nomenclature (ISCN) 2008 [16].

### 2.3. BM Histological Examination

Hematopathologists reviewed Wright-stained BM smears and hematoxylin and eosin- (H&E-) stained sections of BM trephine biopsies for the percentages and patterns of BM infiltration by lymphocytes, lymphoplasmacytic cells, and plasma cells. Immunohistochemical (IHC) staining was performed using CD3, CD20, CD79a, CD138, immunoglobulin *κ*, and immunoglobulin *λ* antibodies (all from Dako, Glostrup, Denmark).

### 2.4. Fluorescence* In Situ* Hybridization

Common chromosomal abnormalities were investigated using commercial FISH probes. We used two probes to detect 6q deletions: A20/PRDM1/SHGC-79576 DNA-FISH probe (Cancer Genetics Italia S.R.I., Milano, Italy) and 6q21/MYC (8q24) dual color (Kreatech Diagnostics, Amsterdam, Netherlands). Other FISH probes, including LSI 13* RB1* (13q14) Spectrum Orange probe, LSI* CDKN2A* (9p21) Spectrum Orange/CEP 9 Spectrum Green probe, TP53/CEP 17 FISH probe mit, LSI 1p36/1q25 probe, and LSI dual-color break-apart probe for* IGH* translocations (all from Abbott Molecular/Vysis, Des Plaines, IL), were used to detect chromosomal abnormalities that are commonly detected in multiple myeloma. Interphase FISH was performed on stored patient BM aspirate specimens. Slides were stained with FISH probes and counterstained with DAPI, and fluorescence signals were then analyzed by fluorescent microscopy (Zeiss, Göttingen, Germany). The results of FISH were recorded according to the ISCN 2008 [17]. The normal cut-off values for the deletion, amplification, or translocation of chromosomal regions were based on the mean (± three standard deviations), and the binomial distribution function [17] of 20 negative controls was analyzed. The cut-off values were 3% for 6q21,* CDKN2A*, and* TP53 *deletions, 4.0% for* RB1* deletion, 1% for 1q amplification, and 2% for* IGH* translocation.

### 2.5. DNA Extraction and Detection of MYD88 L265P Using Sanger Sequencing

Genomic DNA was extracted from frozen BM mononuclear cells from two patients and from the unstained BM slides of 14 patients. DNA was extracted using the MagNA Pure LC DNA Isolation Kit (Roche Applied Science, Indianapolis, IN, USA) according to the manufacturer's instructions. The quality of DNA was analyzed by assessing the 260/280 absorbance ratio using an ND-1000 Spectrophotometer (NanoDrop Technologies, Wilmington, DE, USA).

Two primers (forward, 5′-CTG GCA AGA GAA TGA GGG AAT GT-3′; reverse, 5′-AGG AGG CAG GGC AGA AGT A-3′) were used to amplify a 489-base pair fragment covering the MYD88 L265P site. PCR was performed using 25 ng to 100 ng genomic DNA in 100 *μ*L of PCR solution (10 *μ*L of 10× MG Taq-HF buffer, 0.2 *μ*M of each primer, 10 *μ*L of 2 mM MG dNTPs mixture, 1 *μ*L of MG Taq-HF polymerase (Macrogen Inc., Seoul, Korea), and distilled water). PCR was performed using an initial denaturation step of 5 min at 94°C, followed by 35 cycles of 94°C for 30 s, 62°C for 30 s, and 72°C for 60 s, with a final extension of 7 min at 72°C. The PCR products were purified and sequenced using a BigDye Terminator v3.1 cycle sequencing kit (Applied Biosystems, Foster City, CA) and an ABI 3730 XL automatic sequencer (Applied Biosystems) using the same primers described above.

### 2.6. Statistical Analysis

Fisher's exact test and *χ* ^2^ test were used to compare categorical variables, and the Mann-Whitney *U* test was used for continuous variables. Estimates of overall survival (OS) were made using the Kaplan-Meier method, and differences among survival curves were analyzed using the log-rank test. Cox proportional hazards regression analysis was used to develop a multivariate model of prognostic factors by considering the factors that were associated with survival. Statistical analyses were performed using SPSS version 17.0 (SPSS Inc., Chicago, IL, USA). *P* values < 0.05 were considered statistically significant.

## 3. Results

### 3.1. Clinical and Laboratory Characteristics of Patients

The baseline characteristics of the patients are summarized in Table 1. All patients were Korean, with a median age of 63.5 years (range 40–88 years). There were 17 male (77%) and five female (23%) patients. IgM monoclonal protein was observed in all patients at a median level of 2.52 g/dL (range 0.32–9.74 g/dL). Splenomegaly and lymphadenopathy were observed in 10 (45%) and 16 patients (73%), respectively.

### 3.2. BM Histology

Morphologic examination of BM biopsies and IHC staining revealed diffuse infiltration of CD20+ small lymphoid cells in 11 patients (50%), whereas others presented with patch or nodular infiltration of lymphoid cells. In differential counts of BM aspirations, the median percentage of small and plasmacytoid lymphocytes was 42% (range 3–93%). Four patients presented with <20% of total lymphocytes (14%, 13%, 3%, and 6%). The increase in number of plasma cells was less significant, with a median of 3% (range 0–33%). Three patients presented with >10% of total plasma cells in BM aspirates (13%, 14%, and 33%). Finally, one patient presented with more plasma cells (33%) than total lymphocytes (6%).

### 3.3. Results of a Conventional Cytogenetic Study

Conventional cytogenetic analysis using G-banding was performed in 18 patients. Five patients (28%) exhibited structural abnormalities: three presented with 6q deletions 46,XY,14pstk+[18]/46,XY,idem,i(6)(p10)[2], 46,XY,1qh+,del(6)(q23)[2]/46,XY,1qh+[22], and 45,X,−Y,1qh+,del(6)(q21),inc[3]/45,idem,add(3)(q26.2),der(3)add(3)(p?23)add(3)(q26.2)[13]/46,XY,1qh+[5] as abnormal karyotypes, which were also detected using FISH. Two patients identified as having the* IGH *rearrangement by FISH presented with abnormal karyotypes in G-banding 46,XY,t(1;14)(p?11;q32)[5]/46,XY[4] and 46,XX[14] 46,XX,−1,der(1)add(1)(q42)dup(1)(q21q32)?dup(1)(q32q12),+3,add (14)(q32)[1]/46,XX[19].

### 3.4. Prevalence of 6q Deletion and Other Cytogenetic Abnormalities by FISH

**Figure 1:**
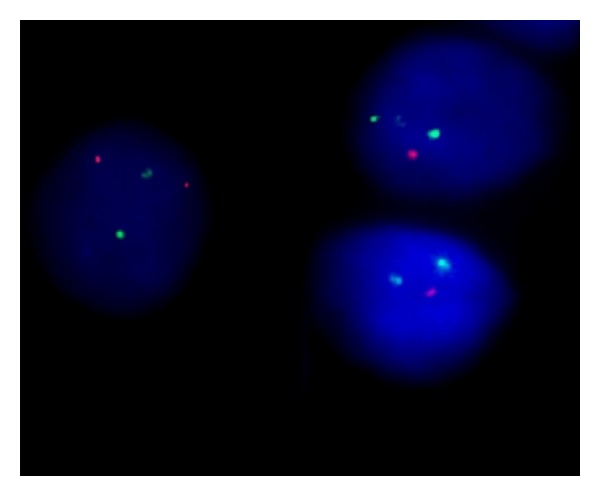
Interphase fluorescence* in situ* hybridization (FISH) analysis of bone marrow using a probe targeting 6q21/8q24. Abnormal cells exhibit one orange (6q21) and two green (8q24) signals, indicating the presence of a 6q deletion.

Among the 22 patients, ten (45%) exhibited cytogenetic aberrations (Table 2). Of these, eight patients (37%) presented with 6q21 deletion (Figure 1). The t(14q32)/*IGH* rearrangement was observed in four patients (18%), amongst whom two patients had both 6q deletion and* IGH* rearrangement, and two patients had only the* IGH* rearrangement.* TP53* deletion was observed in one patient who also had a 6q deletion, and 1q amplification was detected in one patient with an* IGH* rearrangement. The* CDKN2A* (9p21) and* RB1* (13q14) FISH probes did not reveal any abnormalities. Among the ten patients with FISH abnormalities, eight had G-banding data available. Of the six patients with 6q deletions revealed using FISH, three (50%) presented normal karyotypes, whereas other three exhibited concurrent abnormalities by G-banding analysis. Two patients with both the 6q deletion and* IGH* rearrangement by FISH did not exhibit abnormalities at the 14q21 locus by G-banding, and two patients with only the* IGH* rearrangement by FISH presented with concordant abnormalities by G-banding of t(1;14)(p?11;q32) and add(14)(q32), respectively.

### 3.5. MYD88 L265P Mutation and Its Correlation with WM Disease Characteristics

**Figure 2:**
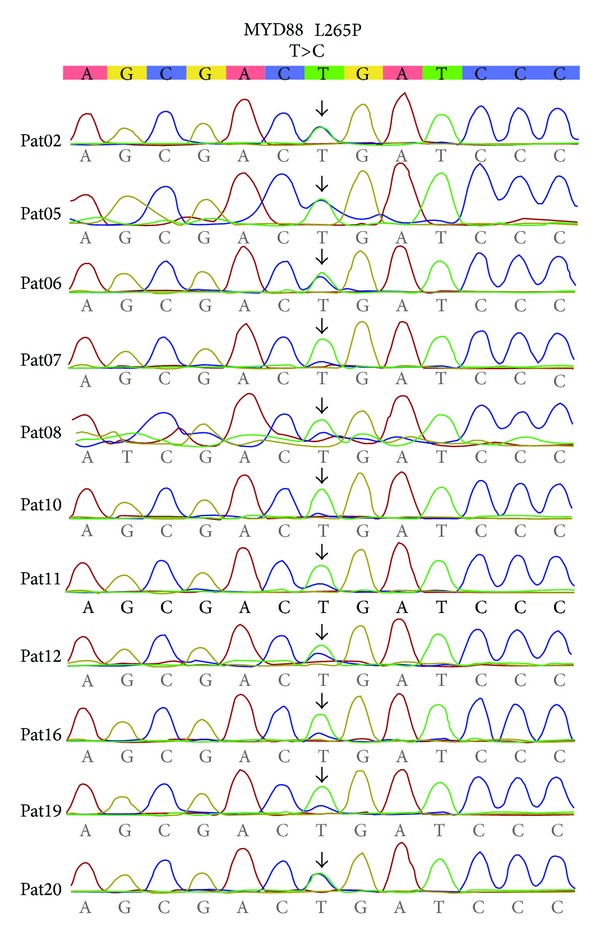
Somatic mutations of MYD88 L265P found in 11 Waldenström macroglobulinemia patients by Sanger sequencing method.

Among the 16 patients for whom the sequencing for* MYD88* gene was possible, 11 (69%) carried the L265P mutation (Figure 2). When* MYD88* mutation-positive and mutation-negative patients were compared, there were no significant differences in the clinical characteristics and IgM monoclonal protein levels between the two groups (Table 3). In addition, there were no significant differences in lymphocyte counts in peripheral blood and BM aspirates. However, when the lymphocyte burden in BM biopsies was compared, most patients with the MYD88 L265P mutation presented with ≥80% of BM cellularity with diffuse infiltration; in contrast, no mutation-negative patients had such a high concentration of lymphocytes in their BM biopsy (*P* = 0.017).

**Figure 3:**
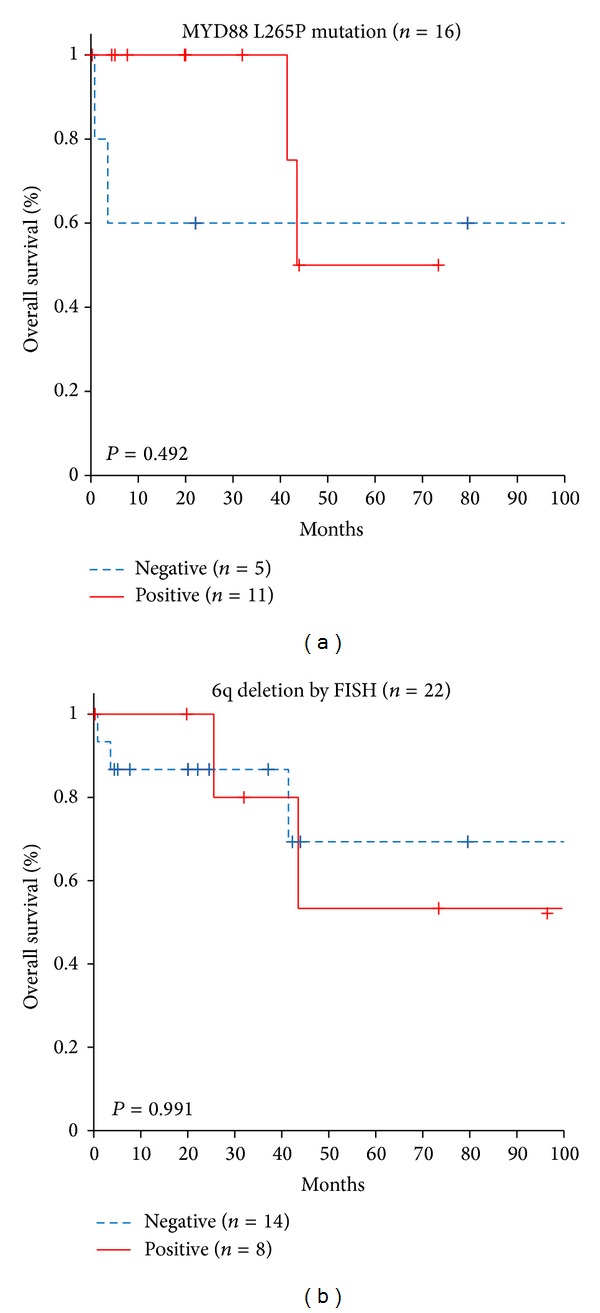
Overall survival of Waldenström macroglobulinemia patients according to the presence of (a) MYD88 L265P mutations and (b) 6q deletion by fluorescence* in situ *hybridization (FISH).

The* MYD88* mutation was found in all six patients with 6q deletion in whom sequencing was possible (*P* = 0.037). There was no significant difference in the presence of the* IGH* rearrangement between mutation-positive and mutation-negative patients (*P* = 0.350). However, neither of the two patients with* IGH* rearrangement without 6q deletion carried a* MYD88* mutation. When the prognosis was compared according to the presence of the MYD88 L265P mutation and 6q deletion, there was no significant difference between patients with and without* MYD88* mutations or 6q deletions (Figure 3).

When we retrospectively reviewed five* MYD88*-negative cases, one patient presented with 5.7% lymphocytes and 32.9% plasma cells in BM aspirates with the* IGH* rearrangement and 1q amplification, suggesting that a diagnosis of IgM plasma cell myeloma might be considered. Additional patients presented with 3.0% lymphocytes and 3.0% plasma cells with no FISH abnormalities, multiple large CD20 lymphoid aggregates, and low levels of monoclonal protein (0.86 g/dL), which might be more consistent with the involvement of BM in diffuse large B cell lymphoma after the review of lymph node biopsies.

## 4. Discussion

This is the first report describing the MYD88 L265P mutation status in Korean WM patients. MYD88 L265P mutation has been commonly reported in recent studies of WM, with frequencies of 70–100%, depending on the method and tissues used for genetic analyses. The MYD88 L265P mutation can also be found in patients with IgM monoclonal gammopathy of unknown significance (IgM MGUS) at a lower frequency than WM, ranging from 10 to 87% [10, 18, 19]. Because the frequency of MYD88 L265P mutation is much lower in other related chronic B cell lymphoproliferative disorders such as splenic marginal zone B cell lymphoma, multiple myeloma, and chronic lymphocytic leukemia (<10%), the presence of this mutation could be a very useful diagnostic marker to distinguish WM from other B cell-related disorders and might represent a potential therapeutic target for WM [18, 19].

In this study, the frequency of the MYD88 L265P mutation was 69% in Korean patients with WM assessed by PCR and Sanger sequencing in unsorted BM cells, which is comparable with a previous study performed in Caucasian patients [9]. Therefore, we confirmed that MYD88 L265P is a major mutation that is also found in most Korean WM patients. When we compared patients with and without the MYD88 L265P mutation, mutation-positive patients tended to exhibit a higher lymphocyte burden on BM biopsy. All the patients enrolled in our study satisfied the WHO criteria, although some mutation-negative patients were borderline between WM and other B-lymphoproliferative disorders or between IgM multiple myeloma and diffuse large B cell lymphoma. Although diagnoses are made by fulfilling the diagnostic criteria for WM, the possibility of overlapping diseases could be inferred in patients without the MYD88 L265P mutation. Clinically, the presence of MYD88 L265P mutation or 6q deletion discriminates WM from IgM MGUS [20]. Future investigation of the MYD88 L265P mutation in overlapping diseases would highlight the role of MYD88 L265P in the pathogenesis of WM and as a potential diagnostic marker.

We also investigated the correlation of cytogenetic aberrations with the MYD88 L265P mutation. The 6q deletion was the most frequent abnormality and was found in 37% of Korean patients. Previous studies performed in Western patients with WM reported the frequency of 6q deletions to be 32–54%, identified using FISH [21–24]. Previously, we reported a lower frequency of 6q deletions (10%) in Korean patients compared with Caucasians [24]. We propose that the previous low frequency of 6q deletion might be due to the small number of patients. The second most frequent abnormality in this study was the* IGH* rearrangement, which was identified in 18% of patients. Half of the patients with the* IGH* rearrangement presented with both 6q deletion and* IGH* rearrangement, whereas the other half presented with only the* IGH *rearrangement.

Interestingly, the 6q deletion was significantly associated with the presence of the MYD88 L265P mutation. All patients with 6q deletions for whom sequencing was possible harbored MYD88 L265P mutations. In contrast, patients with only the* IGH* rearrangement did not present with the MYD88 L265P mutation. The deletion of 6q is the most frequent chromosomal abnormality in WM. Candidate tumor suppressor genes identified in this region are* B lymphocyte-induced maturation protein 1* (*BLIMP1*) and* tumor necrosis factor *α*-induced protein 3 *(*TNFAIP3* or* A20*) [25, 26]. *BLIMP1* is a transcriptional repressor that plays a pivotal role in the differentiation of B cells into plasma cells [27–29]. Therefore, deleting* BLIMP1* would block the differentiation of B cells into plasma cells.* TNFAIP3 *participates in terminating NF-*κ*B signaling, and its loss of function by deletion might enhance inflammatory, autoimmune, and malignant human diseases, including WM [30, 31]. Because* MYD88* is a key player in the activation of the canonical NF-*κ*B pathway, which is downstream of Toll-like receptor and interleukin-1 receptor signaling [32, 33], we hypothesize that the concurrent presence of the MYD88 L265P mutation and deletion of* BLIMP1* or* TNFAIP3* enhance inflammatory reactions that contribute to the pathogenesis of WM.

In previous studies, Jimenez et al. [10] reported that there were no significant differences in 6q deletion between MYD88 L265P mutated and nonmutated groups in IgM-MGUS and WM, although higher mutation rates (84%) were found in patients with 6q deletions. Poulain et al. also reported that the 6q deletion was not significantly associated with MYD88 L265P mutation status [11]. In addition, previous studies revealed some small differences in the clinical and laboratory characteristics of carriers and noncarriers of the mutation, such as lower levels of lymphocytosis and a slightly higher IgM monoclonal component in patients with the mutation [10, 11]. These studies suggest that there are no specific clinical characteristics associated with MYD88 L265P mutation status. Consistent with these observations, we observed no specific differences in most clinical and laboratory parameters between carriers and noncarriers of the MYD88 L265P mutation. We also observed no difference in overall survival, consistent with a previous study [10]. However, we did observe a significantly higher lymphocyte burden in BM biopsy in mutated patients, regardless of peripheral blood lymphocytosis or BM lymphocyte counts. This finding is consistent with the hypothesis that more typical WM disease features occur in patients with the MYD88 L265P mutation.

The limitations of this study are the small number of patients and the use of less sensitive Sanger sequencing to detect the MYD88 L265P mutation. Because WM is a very rare disease, an additional multicenter study should be performed to allow a more comprehensive genetic analysis of Korean WM patients. In addition, studies using different molecular methods and more sophisticated approaches for assessing mutations in minor populations of malignant cells should be performed.

In conclusion, we observed a high incidence of MYD88 L265P mutation and 6q deletion in Korean WM patients. We also found a novel association between MYD88 L265P mutation and 6q deletion, though the small number of patients should be taken into consideration for interpretation of the findings of our study with caution. We suggest assessing MYD88 L265P mutation status and performing cytogenetic studies to characterize 6q deletions in WM could help with the diagnosis of WM. As such, refining the current diagnostic classification system might be attempted based on these novel findings.

## Figures and Tables

**Table 1:** Clinical and laboratory characteristics of 22 patients with Waldenström macroglobulinemia.

Characteristic	Number of patients/total	(%)
Age > 65 years	11/22	50
Male sex	17/22	77
Performance status*		
0 to 2	20/22	91
3 or 4	2/22	9
B symptoms	12/22	55
Hyperviscosity	8/22	36
Cryoglobulinemia	3/10	30
Hepatomegaly	7/22	32
Splenomegaly	10/22	45
Lymphadenopathy	16/22	73
Peripheral neuropathy	2/22	9
Multiple osteolytic lesions	2/22	9
Hemoglobin ≤11.5 g/dL	20/22	91
Platelet < 100 × 10^9^/L	6/22	27
Monoclonal protein type		
IgM*κ*	18/22	82
IgM*λ*	4/22	18

Ig: immunoglobulin.

*Performance status is according to Eastern Cooperative Oncology Group (ECOG) score as follows: 0: without symptoms; 1: mild symptoms not requiring treatment; 2: symptoms requiring some treatment; 3: disabling symptoms but allowing ambulation for >50% of the day; 4: ambulation < 50% of the day.

**Table 2:** Summary of interphase FISH results.

FISH	Number of patients/total	(%)
6q21 deletion	8/22	37
*IGH *(14q32) translocation	4/22	18
*TP53* (17p13) deletion	1/22	5
1q25 amplification	1/22	5
*CDKN2A* (9p21) deletion	0/22	0
*RB1 *(13q14) deletion	0/22	0

FISH: fluorescence *in situ* hybridization.

**Table 3:** Patient characteristics and interphase FISH results according to the presence of MYD88 L265P mutation.

Characteristics	MYD88 L265P (+)	MYD88 L265P (−)	P*
Age ≥ 65 years	6/11 (55)	2/5 (40)	0.590
Male sex	9/11 (82)	3/5 (60)	0.350
B symptoms	7/11 (64)	3/5 (60)	0.889
Hyperviscosity	5/11 (46)	1/5 (20)	0.330
Splenomegaly	5/11 (46)	2/5 (40)	0.839
Lymphadenopathy	7/11 (64)	5/5 (100)	0.120
Multiple osteolytic lesions	1/11 (9)	1/5 (20)	0.541
Hemoglobin ≤11.5 g/dL	10/11 (91)	5/5 (100)	0.486
Platelet < 100 × 10^9^/L	2/11 (18)	2/5 (40)	0.350
Death	2/11 (18)	2/5 (40)	0.350
IgM*κ* type monoclonal protein	10/11 (91)	3/5 (60)	0.142
Monoclonal protein (g/dL)	3.56 (0.50–5.43)	2.94 (0.55–9.74)	0.777
PB lymphocytes (×10^6^/L)	2233 (807–6100)	1415 (1067–21775)	0.955
BM lymphocytes (%)	45.0 (14.4–93.2)	20.7 (3.0–84.7)	0.234
BM plasma cells (%)	3.7 (0–14.0)	3.2 (0–32.9)	0.691
BM cellularity (%)	85 (25–95)	60 (25–85)	0.093
High lymphocyte burden in BM biopsy^†^	7/11 (64)	0/5 (0)	0.017
6q21 deletion	6/11 (55)	0/5 (0)	0.037
*IGH* (14q32) translocation	2/11 (18)	2/5 (40)	0.350
*P53* (17p13) deletion	1/11 (9)	0/5 (0)	0.486
1q25 amplification	0/11 (0)	1/5 (20)	0.126
Abnormal karyotype	3/7 (43)	2/5 (40)	0.921

BM: bone marrow; FISH: fluorescence *in situ* hybridization; Ig: immunoglobulin; PB: peripheral blood.

*P values were calculated using *χ* ^2^ test for categorical variables and Mann-Whitney *U* test for continuous variables between patients with or without the MYD88 L265P mutation.

^†^BM cellularity ≥ 80% with diffuse lymphocytes infiltration in BM biopsy.

## References

[1] Vijay A, Gertz MA (2007). Waldenström macroglobulinemia. *Blood*.

[2] Owen RG, Treon SP, Al-Katib A (2003). Clinicopathological definition of Waldenstrom’s macroglobulinemia: consensus panel recommendations from the Second International Workshop on Waldenstrom’s Macroglobulinemia. *Seminars in Oncology*.

[3] Swerdlow SH, Campo E, Harris NL (2008). *World Health Organization Classification of Tumours of Haematopoietic and Lymphoid Tissues*.

[4] Treon SP, Morel P, Leblond V, Fermand J-P (2005). Report of the Third International Workshop on Waldenstrom's macroglobulinemia. *Clinical Lymphoma*.

[5] Groves FD, Travis LB, Devesa SS, Ries LA, Fraumeni JF (1998). Waldenstrom's macroglobulinemia: incidence patterns in the United States, 1988–1994. *Cancer*.

[6] Ko YH, Kim CW, Park CS (1998). REAL classification of malignant lymphomas in the Republic of Korea. *Cancer*.

[7] Treon SP, Hunter ZR, Aggarwal A (2006). Characterization of familial Waldenstrom's macroglobulinemia. *Annals of Oncology*.

[8] Treon SP, Xu L, Yang G (2012). MYD88 L265P somatic mutation in Waldenstrom's macroglobulinemia. *The New England Journal of Medicine*.

[9] Gachard N, Parrens M, Soubeyran I (2013). IGHV gene features and MYD88 L265P mutation separate the three marginal zone lymphoma entities and Waldenstrom macroglobulinemia/lymphoplasmacytic lymphomas. *Leukemia*.

[10] Jimenez C, Sebastian E, Chillon MC (2013). MYD88 L265P is a marker highly characteristic of, but not restricted to, Waldenstrom's macroglobulinemia. *Leukemia*.

[11] Poulain S, Roumier C, Decambron A (2013). MYD88 L265P mutation in Waldenstrom macroglobulinemia. *Blood*.

[12] Treon SP, Hunter ZR (2013). A new era for Waldenstrom macroglobulinemia: MYD88 L265P. *Blood*.

[13] Varettoni M, Arcaini L, Zibellini S (2013). Prevalence and clinical significance of the MYD88 (L265P) somatic mutation in Waldenstrom's macroglobulinemia and related lymphoid neoplasms. *Blood*.

[14] Xu L, Hunter ZR, Yang G (2013). MYD88 L265P in Waldenstrom macroglobulinemia, immunoglobulin M monoclonal gammopathy, and other B-cell lymphoproliferative disorders using conventional and quantitative allele-specific polymerase chain reaction. *Blood*.

[15] Yang G, Zhou Y, Liu X (2013). A mutation in MYD88 (L265P) supports the survival of lymphoplasmacytic cells by activation of Bruton tyrosine kinase in Waldenstrom macroglobulinemia. *Blood*.

[16] Slovak ML, Campbell LJ International System of Human Cytogenetic Nomenclature.

[17] Slovak ML, Campbell LJ International System of Human Cytogenetic Nomenclature.

[18] Xu L, Hunter ZR, Yang G (2013). MYD88 L265P in Waldenstrom macroglobulinemia, immunoglobulin M monoclonal gammopathy, and other B-cell lymphoproliferative disorders using conventional and quantitative allele-specific polymerase chain reaction. *Blood*.

[19] Varettoni M, Arcaini L, Zibellini S (2013). Prevalence and clinical significance of the MYD88 (L265P) somatic mutation in Waldenstrom's macroglobulinemia and related lymphoid neoplasms. *Blood.*.

[20] Schop RFJ, Van Wier SA, Xu R (2006). 6q deletion discriminates Waldenström macroglobulinemia from IgM monoclonal gammopathy of undetermined significance. *Cancer Genetics and Cytogenetics*.

[21] Terré C, Nguyen-Khac F, Barin C (2006). Trisomy 4, a new chromosomal abnormality in Waldenström’s macroglobulinemia: a study of 39 cases. *Leukemia*.

[22] Schop RFJ, Michael Kuehl W, Van Wier SA (2002). Waldenström macroglobulinemia neoplastic cells lack immunoglobulin heavy chain locus translocations but have frequent 6q deletions. *Blood*.

[23] Chang H, Qi X, Xu W, Reader JC, Ning Y (2007). Analysis of 6q deletion in Waldenstrom macroglobulinemia. *European Journal of Haematology*.

[24] Bang S-M, Seo J-W, Park KU (2010). Molecular cytogenetic analysis of Korean patients with Waldenström macroglobulinemia. *Cancer Genetics and Cytogenetics*.

[25] Gutiérrez NC, Ocio EM, de las Rivas J (2007). Gene expression profiling of B lymphocytes and plasma cells from Waldenström’s macroglobulinemia: comparison with expression patterns of the same cell counterparts from chronic lymphocytic leukemia, multiple myeloma and normal individuals. *Leukemia*.

[26] Poulain S, Braggio E, Roumier C (2011). High-throughput genomic analysis in Waldenström’s macroglobulinemia. *Clinical Lymphoma, Myeloma and Leukemia*.

[27] Alexander Turner C, Mack DH, Davis MM (1994). Blimp-1, a novel zinc finger-containing protein that can drive the maturation of B lymphocytes into immunoglobulin-secreting cells. *Cell*.

[28] Calame KL, Lin K-I, Tunyaplin C (2003). Regulatory mechanisms that determine the development and function of plasma cells. *Annual Review of Immunology*.

[29] Shaffer AL, Shapiro-Shelef M, Iwakoshi NN (2004). XBP1, downstream of Blimp-1, expands the secretory apparatus and other organelles, and increases protein synthesis in plasma cell differentiation. *Immunity*.

[30] Hymowitz SG, Wertz IE (2010). A20: from ubiquitin editing to tumour suppression. *Nature Reviews Cancer*.

[31] Ma A, Malynn BA (2012). A20: linking a complex regulator of ubiquitylation to immunity and human disease. *Nature Reviews Immunology*.

[32] Muzio M, Ni J, Feng P, Dixit VM (1997). IRAK (Pelle) family member IRAK-2 and MyD88 as proximal mediators of IL-1 signaling. *Science*.

[33] Wesche H, Henzel WJ, Shillinglaw W, Li S, Cao Z (1997). MyD88: an adapter that recruits IRAK to the IL-1 receptor complex. *Immunity*.

